# Inactivation of an enterovirus by airborne disinfectants

**DOI:** 10.1186/1471-2334-13-177

**Published:** 2013-04-15

**Authors:** Thomas Thevenin, Pierre-Emmanuel Lobert, Didier Hober

**Affiliations:** 1 Laboratory of Virology/EA3610, University Lille 2, Faculty of Medecine, CHRU Lille, Institut Hippocrate, 152 rue du Dr Yersin, Loos-Lez-Lille, 59120, France

## Abstract

**Background:**

The activity of airborne disinfectants on bacteria, fungi and spores has been reported. However, the issue of the virucidal effect of disinfectants spread by fogging has not been studied thoroughly.

**Methods:**

A procedure has been developed to determine the virucidal activity of peracetic acid-based airborne disinfectants on a resistant non-enveloped virus poliovirus type 1. This virus was laid on a stainless carrier. The products were spread into the room by hot fogging at 55°C for 30 minutes at a concentration of 7.5 mL.m^-3^. Poliovirus inoculum, supplemented with 5%, heat inactivated non fat dry organic milk, were applied into the middle of the stainless steel disc and were dried under the air flow of a class II biological safety cabinet at room temperature. The Viral preparations were recovered by using flocked swabs and were titered on Vero cells using the classical Spearman-Kärber CPE reading method, the results were expressed as TCID50.ml^-1^.

**Results:**

The infectious titer of dried poliovirus inocula was kept at 10^5^ TCID_50_.mL^-1^ up to 150 minutes at room temperature. Dried inocula exposed to airborne peracetic acid containing disinfectants were recovered at 60 and 120 minutes post-exposition and suspended in culture medium again. The cytotoxicity of disinfectant containing medium was eliminated through gel filtration columns. A 4 log reduction of infectious titer of dried poliovirus inocula exposed to peracetic-based airborne disinfectant was obtained.

**Conclusion:**

This study demonstrates that the virucidal activity of airborne disinfectants can be tested on dried poliovirus.

## Background

It has been put forward that a thorough disinfection of rooms (air and surface), in a hospital environment, can be achieved thanks to hydrogen peroxide [1]. Bacterial disinfection can be obtained through fogging of product as already proved [2]. The bactericidal, fungicidal and sporicidal activities of aerial surface disinfection can be tested thanks to described procedures [3].

The transmission of viruses through contaminated surfaces has been described [4]. The resistance of viruses to biocides depends on their size, structure and on the presence of a viral envelope [5]. Non-enveloped viruses are in general more resistant to disinfection and drying than enveloped viruses. Without proper disinfection, the infectivity of viruses can be maintained a few hours in the case of enveloped viruses and up to 60 days for non-enveloped viruses [6].

The virucidal activity of liquid products towards viruses in suspension and viruses dried on a surface has been described and procedures have been recommended [7]. In contrast, papers reports the virucidal effect of airborne disinfection are sparse [8-12].

Based on procedures aimed to determine the bactericidal, fungicidal and sporicidal activities of products spread by aerial way for the disinfection of surfaces, we investigated the effect of peracetic acid-based airborne disinfectants toward poliovirus type 1, a model of non-enveloped resistant virus.

## Methods

### Virus and cells

The poliovirus type 1 strain SABIN LSc-2ab was obtained from the Eurovir Hygiene-Institut (Luckenwalde, Germany).

The Vero (ATCC CCL-81) cell line was obtained from the European Collection of Cell Cultures (ECACC) (Sigma-Aldrich, L’isle d’Abeau Chesnes, France).

The poliovirus was spread in a flask of Vero cells cultured in DMEM (Dulbecco’s Modified Eagle’s Medium, Invitrogen, France) supplemented with 2% FBS (Fetal Bovine Serum), 1% Non Essential Amino Acids and 1% L-glutamine at 37°C in a 5% CO_2_ atmosphere. When a cytopathic effect of at least 90% appeared, the cells were scratched off and the cell suspension was freezed (−80°C) and thawed three times and then centrifuged at 1000 g for 10 minutes. The resulting supernatant was aliquoted and stored at −80°C.

Viral preparations were titered on Vero cells using the classical Spearman-Kärber CPE reading method, the results were expressed as TCID_50_.ml^-1^[13].

### Airborne disinfection

Autoclaved stainless disc (20 mm diameter with grade 2B finish on both sides, provided by DEVOS SA, Lille, France) was suspended vertically with dried inocula oriented at the opposite side of the emission source (Aerosept compact 250 (Laboratoires Anios®)) placed in an airtight room (71 m^3^). The stainless disc was placed at a distance of 3.9 m of the emission source, at a height of 1.05 m. Aseptanios Oxy + (Peracetic acid, 1260 ppm) and Aseptanios AD (Peracetic acid, 2500 ppm) are ready-to-use solutions manufactured and provided by Laboratoires Anios® (Lille-Hellemes, France). The products were diffused by hot fogging at 55°C in the room for a duration of 30 minutes at a concentration of 7.5 mL.m^-3^.

50 μL of poliovirus inoculum, supplemented with 5%, heat inactivated (30 min, 105°C) non fat dry organic milk (100 g.L^-1^), was applied in the middle of each stainless steel disc and these discs were dried under the air flow of a class II biological safety cabinet at room temperature (20 ±2°C) for 2 h (±15 min) with a relative humidity from 50 to 75%. The inocula were recovered from discs thanks to wet flocked swabs (COPAN), 60 and 120 minutes after the end of the product diffusion, i.e. 90 and 150 min after drying. The swabs were vortexed for 15 seconds in 1 mL of culture medium and the fluids were harvested.

### Gel fitration

Gel filtration was used in order to eliminate the cytotoxicity of fluids containing disinfectant. TE buffer (pH 7.6) was used to wash the Sephacryl® (S-400 High Resolution, Healthcare GE) slurry three times, each time a centrifugation of one minute at 700 g was done. Empty poly-prep® chromatography columns (BioRad®) were then filled with 10 mL of the washed Sephacryl® slurry and centrifugated one minute at 700 g to eliminate the washing buffer. One mL of the tested preparation could then be filtrated by adding it to the prepared column and centrifugating them for 1 minute at 700 g. The flow-through was then titered as described in “Virus and cells” (see above).

## Results

### Poliovirus dried on stainless carriers

50 μL of poliovirus inoculum applied in the middle of each stainless steel disc were dried under the airflow of a class II biological safety cabinet at room temperature (20 ±2°C) for 2 h (±15 min). After 0, 90 and 150 min, the dried inoculum was recovered and titrations were performed as described in the Materials and Methods section.

**Figure 1:**
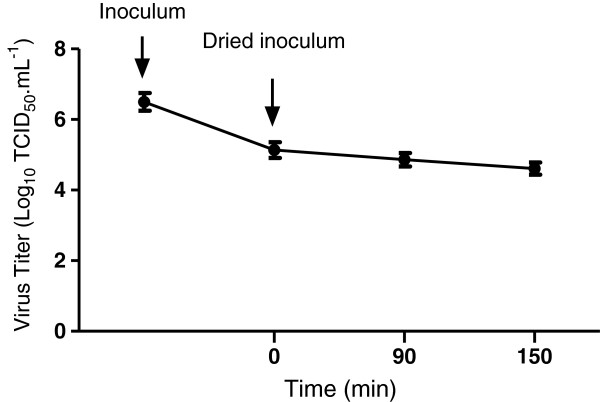
**Infectious titers of poliovirus inocula dried on stainless steel carriers.** An inoculum of 50 μL of culture supernatant fluid containing poliovirus was applied on stainless carriers in duplicate. It was dried and thereafter recovered by using wet flocked swabs at 0, 90 or 150 min. The infectious titers were determined and expressed as Log_10_ TCID_50_.mL^-1^. The results are the mean +/- S.D of three independents experiments.

As shown in Figure 1, the infectious titer of inoculum before drying was 10^6^ TCID_50_.mL^-1^ . After drying, it was 10^5.1^ TCID_50_.mL^-1^ and was roughly maintained at this level up to 150 minutes after drying (10^4.6^ TCID_50_.mL^-1^).

### Poliovirus inactivation with airborne disinfectants

The resistance of poliovirus to drying prompted us to test the virucidal activity of airborne disinfectants towards this virus according to a procedure derived from the NF T 72–281 norm [7]. Poliovirus inocula were exposed to disinfectants (Aseptanios AD and Aseptanios Oxy+) for 30 min.

Due to the cytotoxicity of peracetic acid-based disinfectants (up to 1:1,000 dilution), the measurement of the infectious titer of recovered inocula that were exposed to disinfectant products was impaired (data not shown). Gel filtration columns were used to eliminate (3 log reduction) the cytotoxicity of the products (data not shown).

**Figure 2:**
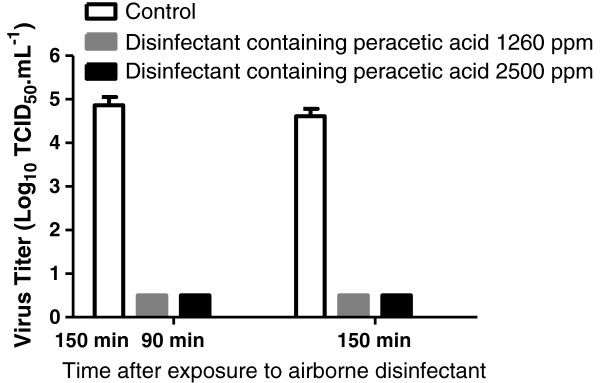
**Infectious titers of dried poliovirus inocula after exposure to airborne disinfectant.** 50 μL of culture supernatant fluids containing poliovirus supplemented with 5% milk were applied on stainless carriers in duplicate. They were dried and afterwards exposed to airborne disinfectant products containing peracetic acid 1260 ppm (ASEPTANIOS AD) and 2500 ppm (ASEPTANIOS OXY+) or not exposed (control). The disinfectant products were diffused in the room during 30 minutes. Thereafter 60 and 120 min following the diffusion of disinfectants (90 and 150 min after drying respectively), the carrier surface was scrubbed with a wet flocked swab to recover the iconulum that was then resuspended in culture medium (1 mL) again. The medium was filtrated through a column to eliminate the cytotoxic disinfectant products. Afterwards the infectious titer was determined. The results, expressed as Log_10_ TCID_50_.mL^-1^, are the mean + S.D of two experiments.

The procedure of gel filtration of virus samples recovered 90 and 180 minutes after drying did not reduce the infectious titers (data not shown). After gel filtration, it was demonstrated that the infectious titers of inocula exposed to both disinfectants were dramatically reduced to at least 4 log_10_ (see Figure 2).

Whether the low virus titers (less than the treshold value) in samples exposed to airborne disinfectants in our experiments were the result of an inactivation of viral particles on the stainless steel carrier or in suspension in culture medium containing disinfectants after recovering was investigated. On the one hand, the recovered viral particles were in suspension together with disinfectants for a very short period of time (less than 30 s) before gel filtration (duration of 1min), and on the other hand the incubation of viral particles in suspension in presence of a disinfectant (Aseptanios AD or Aseptanios Oxy+) for 30 s resulted in a moderate reduction of infectious titres (1.5 log) only, whereas a longer incubation time (5 min) was needed to obtain a 4 log_10_ reduction of the infectious titer (data not shown).

## Discussion

Airborne disinfection is an efficient disinfecting method for various surfaces to inactivate bacteria, fungi and spores [1,14]. However, the efficiency of airborne disinfection against viruses was not extensively reported. Therefore we decided to take advantage of procedures described in a norm [3] recommended for testing airborne disinfection against microbes (bacteria, fungi and spores) to evaluate the virucidal effect of disinfectants diffused by hot fogging on poliovirus type 1, a non enveloped virus which is resistant to disinfectants in suspension.

The virucidal activity of peracetic acid (PAA) on enveloped and non-enveloped viruses (duck hepatitis B virus, vaccinia virus, adenovirus type 2, poliovirus type 1) in suspension, and on adenovirus or murine norovirus on stainless steel carrier was reported [15,16]. For the first time, the present work demonstrates, that airborne peracetic acid-based disinfectants can inactivate dried poliovirus inocula on stainless steel carrier as well.

Our data obtained in conditions of humidity and temperature similar to those that were previously recommended or reported [3,9], are in agreement with those of other groups who recently reported that vapours of hydrogen peroxide, which together with peracetic acid are peroxygen, had virucidal activity on various viruses, especially poliovirus [8,9]. The advantage of peracetic acid is that its biocidal effect, higher than the one of hydrogen peroxide, is obtained at low concentrations even in presence of soils (organic or inorganic) [17].

Peracetic acid is a broad-spectrum disinfectant that showed a virucide activity on enveloped and non-enveloped viruses [16,18,19]. The activity of peracetic acid is similar to the peroxygen, it denatures proteins and enzymes and increases the permeability of membranes by disrupting sulfhydryl and sulphur bonds [15]. It is an oxiding agent that has probably direct effects on viral proteins and RNA and indirect effects through the production of hydroxyl radicals and other short-lived products [20].

In a hospital environment, stainless steel is used for door handles, hand and grab rails in toilets. Therefore stainless steel discs, which were used in this study, were relevant to test the efficiency of disinfectants on surfaces. Furthermore these discs do not bind, absorb or sequester viruses and are small enough to be easily handled, as it was previously described [7].

When virus preparations were exposed to peracetic acid, the recovered samples were cytotoxic due to the presence of biocide, which impaired the measurement of virus titers. Therefore, the elimination of disinfectants from the recovered samples is critical before testing the level of infectious viral particles in recovered samples. Gel filtration columns enabled us to remove the disinfectants and therefore the cytotoxicity of samples without affecting the titer of poliovirus. This is in agreement with results of previous studies that demonstrated the efficiency of such a methodology for this purpose when disinfectant products were tested against viruses in suspension and on surfaces [9,21,22].

In this study, flocked swabs were used to recover viruses on stainless steel discs. Flocked swab has already been used to recover infectious agents from various surfaces [23]. The recovering of virus with flocked swabs, as described in the present study, opens up the possibility to test the virucidal effect of airborne disinfectants on viruses applied on various surfaces regardless of their size.

## Conclusions

Overall the present study brings a proof of principle. A system based on the combination of virus recovering by using flocked swabs and disinfectant elimination through gel filtration columns has the potential of being used for evaluating the virucidal effect of airborne disinfectants. This system displays that hot fogging with peracetic-based disinfectant can reduce poliovirus titres on surfaces.

## Competing interests

The authors declare that they have no competing interests.

## Authors’ contribution

TT and P-EL carried out the experiments together. TT analyzed the data and drafted the manuscript. DH designed and managed the study and participated in the analysis of data and the preparation of the manuscript. All authors read and approved the final manuscript.

## Pre-publication history

The pre-publication history for this paper can be accessed here:

http://www.biomedcentral.com/1471-2334/13/177/prepub
